# Myocardial hypertrophy reduces transmural variation in mitochondrial function

**DOI:** 10.3389/fphys.2014.00178

**Published:** 2014-05-07

**Authors:** Premi Haynes, Kenneth S. Campbell

**Affiliations:** Department of Physiology, Center for Muscle Biology, University of KentuckyLexington, KY, USA

**Keywords:** transmural, hypertrophy, mitochondria, heart failure, ventricular function

There is growing evidence that some cellular properties of mammalian hearts are transmurally heterogeneous, varying systematically from the inner (sub-endocardium) to the outer (sub-epicardium) region of the left ventricular wall. For example, action potential duration (Lou et al., 2011), calcium sensitivity (Cazorla et al., 2005; Haynes et al., 2014), mitochondria with faster sedimentation rate (Whitty et al., 1976), and β myosin heavy chain isoform (Stelzer et al., 2008) are significantly greater in the sub-endocardium than in the sub-epicardium of the left ventricular wall. Transmural differences in the phosphorylation of myosin light chain-2 (Davis et al., 2001), the dynamics of Ca^2+^ handling and contraction (Campbell et al., 2013), and myocyte orientation (Streeter et al., 1969; Schmid et al., 2005) have also been shown. These heterogeneities may be important for ventricular function (Ingels, 1997; Sengupta et al., 2006). For example, ventricular torsion (the wringing motion of the heart) augments systolic ejection and has been linked to transmural heterogeneities in myocardial architecture, action potential duration, and contractile properties (Streeter et al., 1969; Evangelista et al., 2011; Campbell et al., 2013).

Data from several labs now show that transmural variation in cellular-level properties can be disrupted in diseased human (Lou et al., 2011; Haynes et al., 2014) and animal (Humphrey et al., 1988; Cazorla et al., 2005) hearts. This raises a fundamental question. Does the loss of transmural variation cause the disease, or is it a consequence of remodeling? One way of answering this question is to determine how transmural heterogeneity changes during the development of cardiac disease. These data might ultimately help the field to develop better therapies for heart failure.

The recent study by Kindo et al. (2012) in this journal investigated whether transmural variation in mitochondrial function precedes heart failure. The authors induced mild left ventricular hypertrophy in rats by banding the abdominal aorta for 6-weeks to induce pressure overload. The treated rats did not show clinical symptoms of heart failure (depressed ejection fraction and fractional shortening) but exhibited clear mitochondrial dysfunction when compared to the sham animals. One of the important findings was that in the sham animals, the sub-epicardial tissue had ~55% greater mitochondrial respiratory chain complex IV activity than the sub-endocardium. This transmural gradient was reduced in the rats that had been subjected to pressure overload. Specifically, complex IV activity was lower in the sub-epicardium of these animals, which suggests that the sub-epicardium was more affected by the remodeling.

Dysfunction in complex IV activity of the electron transport chain can disrupt the proton gradient needed for ATP synthesis and may compromise energy dependent processes including cross-bridge cycling, and the pumping of Ca^2+^ ions (Carley et al., 2014). Although previous studies have shown that heart failure is associated with mitochondrial dysfunction (Rosca et al., 2011; Carley et al., 2014), the work of Kindo et al. (2012) is the first to show that transmural region-dependent mitochondrial dysfunction precedes overt ventricular failure. These new data are important and augment prior studies that have focused primarily on ischemic tissue. For example, a study by Humphrey et al. (1988) showed that after 25 min of global ischemia the myocytes from the sub-endocardium had lower ATP levels than myocytes from the sub-epicardium. Another study investigated a long term effect of ischemia in rat hearts by ligating a coronary artery and examining the animals after 12 weeks. The activities of complex I and complex IV were decreased in the sub-endocardial tissue (Andre et al., 2013). These two studies are particularly interesting because they suggest that ischemia may produce the biggest detriments in sub-endocardial issue. In contrast, Kindo et al. (2012) studied non-ischemic remodeling and showed that sub-epicardium was more affected. The sensitivity of the sub-epicardium to adaptations prior to heart failure is further supported by data that describe relaxation dynamics in myoctyes isolated from Fisher 344 rats of different ages. Cells from the sub-epicardium showed greater age-dependent changes in relaxation dynamics than cells from other regions (Campbell et al., 2013). One possibility is that ischemic and non-ischemic remodeling produces different transmural effects. However more data are clearly required to test this hypothesis.

Myocytes in the sub-epicardium and sub-endocardium are aligned close to the base to apex axis while myocytes in the mid-myocardium (middle transmural region) are circumferentially arranged (Streeter et al., 1969; Greenbaum et al., 1981). A recent study by Haynes et al. (2014) showed that isometric force is higher in the mid-myocardium than in the sub-epicardium and sub-endocardium of non-failing humans hearts. Most of this transmural variation was lost in diseased human organs. Cazorla et al. (2005) performed similar experiments using rat tissue but did not show significant transmural effects in force production. Other studies in pigs (Stelzer et al., 2008; Van Der Velden et al., 2011) have only investigated the sub-epicardium and the sub-endocardium. Dissecting the ventricular wall into two, as opposed to three or more sections, may hide important transmural effects as myocytes arranged in orthogonal directions may undergo different stress patterns during the cardiac cycle. A definitive test of this hypothesis probably requires analyzing samples from multiple transmural regions from many locations along the base to apex axis. However, this would require large numbers of experiments and a design that could be hard to reproduce in different labs because of its complexity.

The importance of the findings reported by Kindo et al. (2012) reinforce the significance of documenting the anatomical source of myocardial samples that are used in basic science experiments. Transmural variation is also likely to be important in clinical settings. For example, Wachtell et al. (2010) have shown that fractional shortening of the middle transmural region is a better predictor of clinical endpoints than the shortening of other regions, or than traditional global measures of ventricular function such as ejection fraction. Improved understanding of the transmural variation that can contribute to these effects may help scientists and clinicians to develop better therapies for patients with heart disease. The recent work by Kindo et al. is an important step in this process.

## Conflict of interest statement

The authors declare that the research was conducted in the absence of any commercial or financial relationships that could be construed as a potential conflict of interest.
